# Pyrolytic and Kinetic Characteristics of the Thermal Decomposition of *Perilla frutescens* Polysaccharide

**DOI:** 10.1371/journal.pone.0052597

**Published:** 2012-12-26

**Authors:** Quancheng Zhou, Guihua Sheng

**Affiliations:** 1 School of Agricultural Engineering and Food Science, Shandong University of Technology, Zibo, China; 2 School of Life Science, Shandong University of Technology, Zibo, China; National Cancer Institute at Frederick, United States of America

## Abstract

The thermal decomposition of *Perilla frutescens* polysaccharide was examined by thermogravimetry, differential thermogravimetry, and differential thermal analysis. The results showed that the mass loss of the substance proceeded in three steps. The first stage can be attributed to the expulsion of the water from ambient temperature to 182°C. The second stage corresponded to devolatilization from 182°C to 439°C. The residue slowly degraded in the third stage. The weight loss in air is faster than that in nitrogen, because the oxygen in air accelerated the pyrolytic reaction speed reaction. The heating rate significantly affected the pyrolysis of the sample. Similar activation energies of the degradation process (210–211 kJ mol^−1^) were obtained by the FWO, KAS, and Popescu techniques. According to Popescu mechanism functions, the possible kinetic model was estimated to be Avrami–Erofeev 20 *g*(*α*) = [−ln(1–*α*)]^4^.

## Introduction

*Perilla frutescens* (L.) Britton is an annual herbaceous plant belonging to the Labiatae family [1]. *P. frutescens* is an important cash crop; its flowers, leaves, stems, and fruits are highly valuable [2], [3]. The dry ripe fruits of *P. frutescens* and perilla seeds are rich in oil, having an oil content of 36%–50%, which is higher than cottonseed, rapeseed, and castor seed [4].

After oil manufacture, seed cakes are mainly treated as wastes and abandoned or sold as feed because their use has not been fully studied or developed [5]. Seed cakes such as rapeseed cake, safflower cake, cottonseed, and soybean cake have been investigated as thermal decomposition materials for the production of bio-oil and chemical products [6]–[10]. However, the pyrolysis of *P. frutescens* cake has not been studied. The composition and content of a polysaccharide significantly influence its thermal decomposition. Polysaccharides are the major constituents of biomass. With the growing interest in utilizing bio-oil obtained from fast pyrolysis of biomass for fuels and chemicals, understanding the polysaccharide pyrolysis behavior has gained particular importance. Therefore, the thermal decomposition characteristics of the polysaccharide content of *P. frutescens* cake need to be studied. Polysaccharides are commonly found in biological organisms and exhibit bioactivities such as immunity enhancement, anti-tumor, anti-inflammation, and anti-ulcer properties [11], [12]. Hence, a study on the thermal stability of *P. frutescens* polysaccharide, a potential macromolecular drug, is considerably important. The thermal and kinetic analyses are also essential for the identification of polysaccharides and their three-dimensional structure [3]–[7]. Previous studies have investigated the thermal cracking characteristics of cellulose [13]–[17], hemicelluloses [18]–[20], and lignin [21]–[23]. However, no study has focused on such characteristics of *P. frutescens* polysaccharide. Hence, thermogravimetry (TG) and other methods are adopted in the current work to analyze the thermal characteristics and kinetics of *P. frutescens* polysaccharide. The thermal stability and thermal decomposition of the substance are discussed. A reference for the thermal decomposition of seed cakes and development of polysaccharides is also provided.

## Materials and Methods

### Experimental Materials

*P. frutescens* seeds were purchased from the Boshan Market in Zibo. *P. frutescens* polysaccharide was prepared in our laboratory according to the following process: raw *P. frutescens*→pulverization→40 mesh sieving→de-oiling with 60–90°C petroleum ether→80°C water extraction for 4 h→concentration with rotary evaporator→deposition with 70% alcohol→freeze drying→*P. frutescens* polysaccharide.

### Instruments

A TG/DSC STA449C-QMS403C thermal analyzer manufactured by NETZSCH Company (Germany) was used.

### Experiment

*P. frutescens* polysaccharide weighing 20 mg was placed in an alumina crucible. The substance was subjected to TG, differential TG (DTG), and differential thermal analysis (DTA) in air and nitrogen atmosphere, under the following conditions: flow rate of 80 mL min^−1^; heating rates of 10, 30, and 50°C min^−1^; and from room temperature to 800°C.

## Results and Discussion

### Influence of different atmospheres on the thermal decomposition of *P. frutescens* polysaccharide

**Figure 1:**
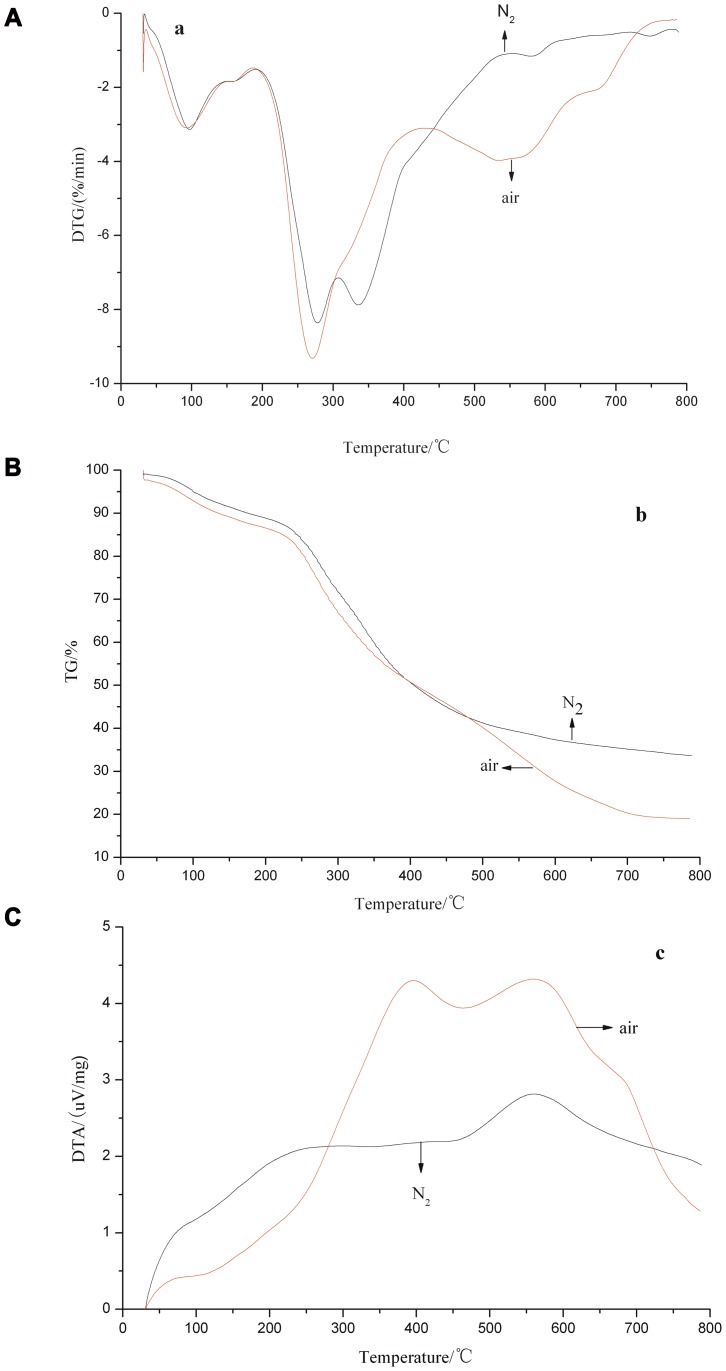
Pyrolytic curves of *Perilla frutescens* polysaccharide under different atmospheres. (a) DTG, (b) TG, and (c) DTA.

Figures 1A–C illustrate the thermal decomposition of *P. frutescens* polysaccharide in air and nitrogen. Similar TG and DTG curves are obtained. Absorbed water is lost in the first stage under both atmospheres, with temperatures ranging from room temperature to 189 and 191°C, respectively, with corresponding total weight losses of 13% and 11% as well as maximum weight losses of 5.55% and 6.03% at 96 and 97°C, respectively. The curves are similar for air and nitrogen without weight loss differences, although the weight loss starts 2°C earlier in air than in nitrogen. These conditions demonstrate that the weight loss is independent of the type of atmosphere in this stage and does not depend on the substances being combusted. As shown in the DTA diagram, more thermal discharges occur in nitrogen. The weight loss in the second stage, which corresponds to 189–432 and 190–554°C, is mainly caused by the decomposition of volatile substances and the polysaccharide. The weight losses in air and nitrogen are fastest at 273 (25.2%) and 278°C (22.8%), respectively. In this stage, a considerable difference between the total weight losses of the polysaccharide under the two atmospheres is found (52.8% vs. 60.9%). This finding shows that a fierce decomposition reaction occurs in P. frutescens polysaccharide within this temperature range, and that this app:ds:perillapolysaccharide is comparatively stable below 190°C. The weight loss in air is faster than that in nitrogen, with more abundant and intense thermal discharges, because the oxygen in air is involved in the decomposition reaction and greatly accelerated the pyrolytic reaction speed reaction in air. This phenomenon was consist with conclusion in reference [24]. Consequently, thermal discharge by the combustion of *P. frutescens* polysaccharide occurs. The total weight loss of *P. frutescens* polysaccharide is higher in nitrogen because of the wider temperature range for decomposition than that in air. The weight loss within the same temperature range under nitrogen decomposition is 53.3%, which is smaller than that in air. The wider temperature range of the decomposition in nitrogen is based on the slower volatilization or evaporation of weightless substances with low boiling points. The third stage involves the slow decomposition of the remaining materials, generating porous residues. The masses of final residues in air and nitrogen are 19.0% and 33.6%, respectively. In air, a rapid weight loss in *P. frutescens* polysaccharide appears at 537°C with a weight loss of 64.1%, which is smaller than that under nitrogen environment at 305°C. This phenomenon is due to the fact that *P. frutescens* polysaccharide intensely burns in air and causes residue polymerization, which covers the surface of materials and hinders the volatilization of internal substances. With increased temperature, the substances on the surface are depolymerized, which results in another remarkable weight loss. Therefore, *P. frutescens* polysaccharide is more stable in nitrogen. A similar phenomenon was also reported in other literature [25].

As shown in Figure 1, two peaks representing thermal discharges can be found in the pyrolysis of *P. frutescens* polysaccharide in air at 398 and 562°C, in contrast to the thermal discharge peak in nitrogen at 560°C. The thermal discharge in air is higher than that in nitrogen because the thermal discharge in air is based on oxidative combustion, whereas that in nitrogen depends on the volatilization and evaporation of substances or cracking of the polysaccharide. These phenomenons were consist with conclusions in reference [24]. The DTA peaks are smaller than the DTG peaks, which indicate that the thermal discharge reaches the maximum at the maximum rate of mass loss, during which the substances start to absorb heat. The increase in temperature, substance decomposition, as well as escape and thermal discharge of the products lead to the maximum values for the rate of mass loss and thermal discharge. The escape of the products decreases with significantly decreased pyrolysis products, slower mass loss of substances, polymerization of pyrolysis products, and thermal absorption. Consequently, the DTA peak flattens.

### Comparison of the pyrolysis characteristics under different heating rates

**Figure 2:**
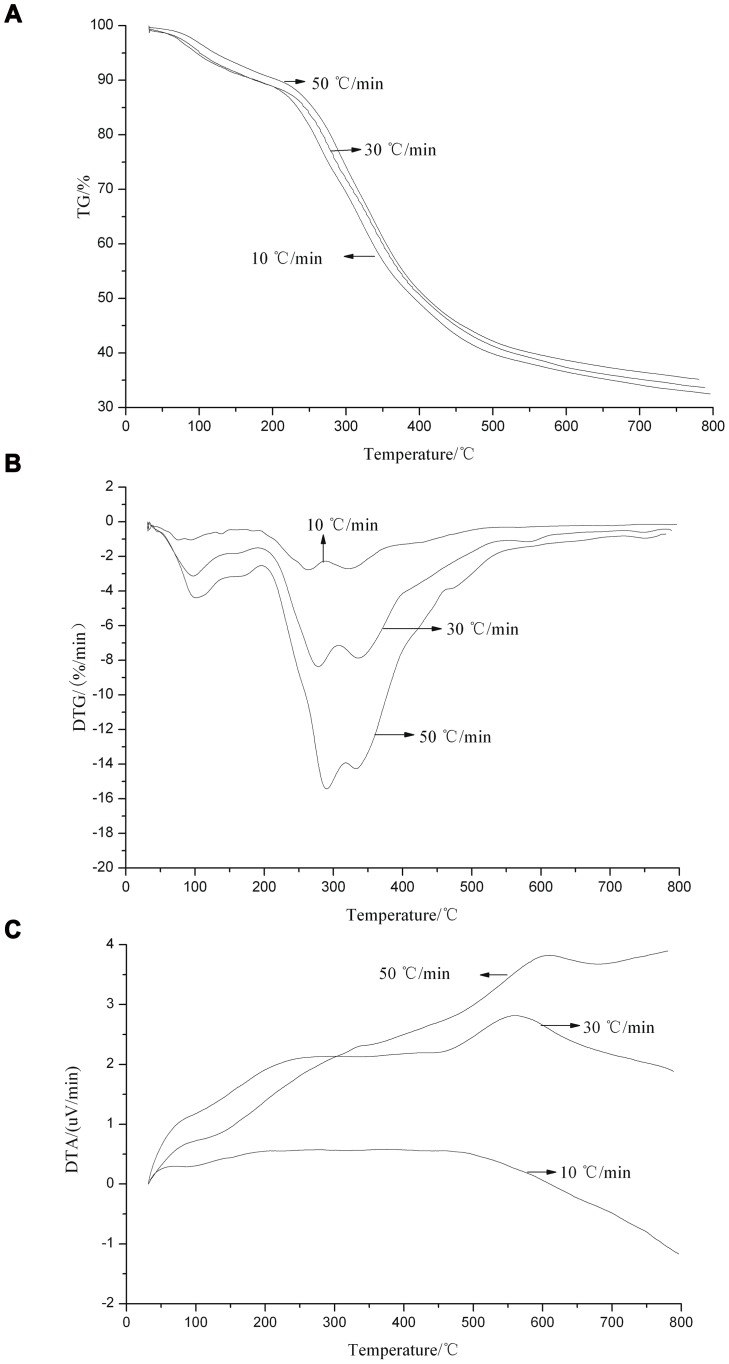
Pyrolytic curves of *Perilla frutescens* polysaccharide at different heating rates. (a) TG, (b) DTG, and (c) DTA.

As shown in Figures 2A–C, the starting temperature for pyrolysis in stages 1 and 2, the temperature for the maximum mass loss rate, and the maximum mass loss rate are all enhanced with increased heating rate. The temperatures for the maximum mass loss rates are 263, 278, and 290°C corresponding to the heating rates of 10, 30, and 50°C min^−1^. These phenomena are caused by the higher thermal inertias at higher heating rates. The temperature difference is larger for higher thermal discharge value, consistent with the maximum and second highest thermal discharges at 50 and 30°C min^−1^, respectively, in the DTA curve. Hence, the heating rate significantly influences the pyrolysis of *P. frutescens* polysaccharide. As shown in Table 1, *P. frutescens* polysaccharide exhibits better thermal stability than other carbohydrates, with a higher residue mass at 800°C. The amount of residues and peak of the second stage differ among various carbohydrates. This finding is related to the source and type of polysaccharide.

**Table 1:** Decomposition temperatures and residues of seven carbohydrates [11], [12].

Sample	TG peak temperature (°C)	Mass ratio of residues at 800°C
*Perilla frutescens* polysaccharide	263	32.5
Abalone polysaccharide	282	29.5
*Ilex* holly leaf polysaccharide	286	28.7
Heparin	267	22.5
Starch	309	17.1
Galactose	308	14.9
Chitin	350	16.5

The heating rate in nitrogen atmosphere is 10°C min^−1^.

### Thermal analysis kinetics

The FWO [26]–[30], KAS [31]–[32], and Popescu methods [27]–[29] were used to determine the parameters of thermal analysis kinetics and analyze the 41 kinetic mechanisms of Popescu (skipping the functional equation and results) [32].

The FWO equation is written as
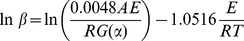
(1)The KAS equation is written as
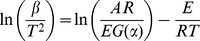
(2)The Popescu equation is written as
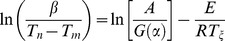
(3)

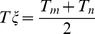
(4)where *β* is the linear heating rate (°C•min^−1^); *E* is the activation energy; (J•mol^−1^); *A* is the pre-exponential factor; *R* is the molar gas constant; *g*(*α*) is the integral mechanism function; and *T*, *T_n_*, *T_m_*, and *T_ξ_* are temperatures at the time of n, m and ξ.

### Determination of the parameters of thermal analysis kinetics

**Figure 3:**
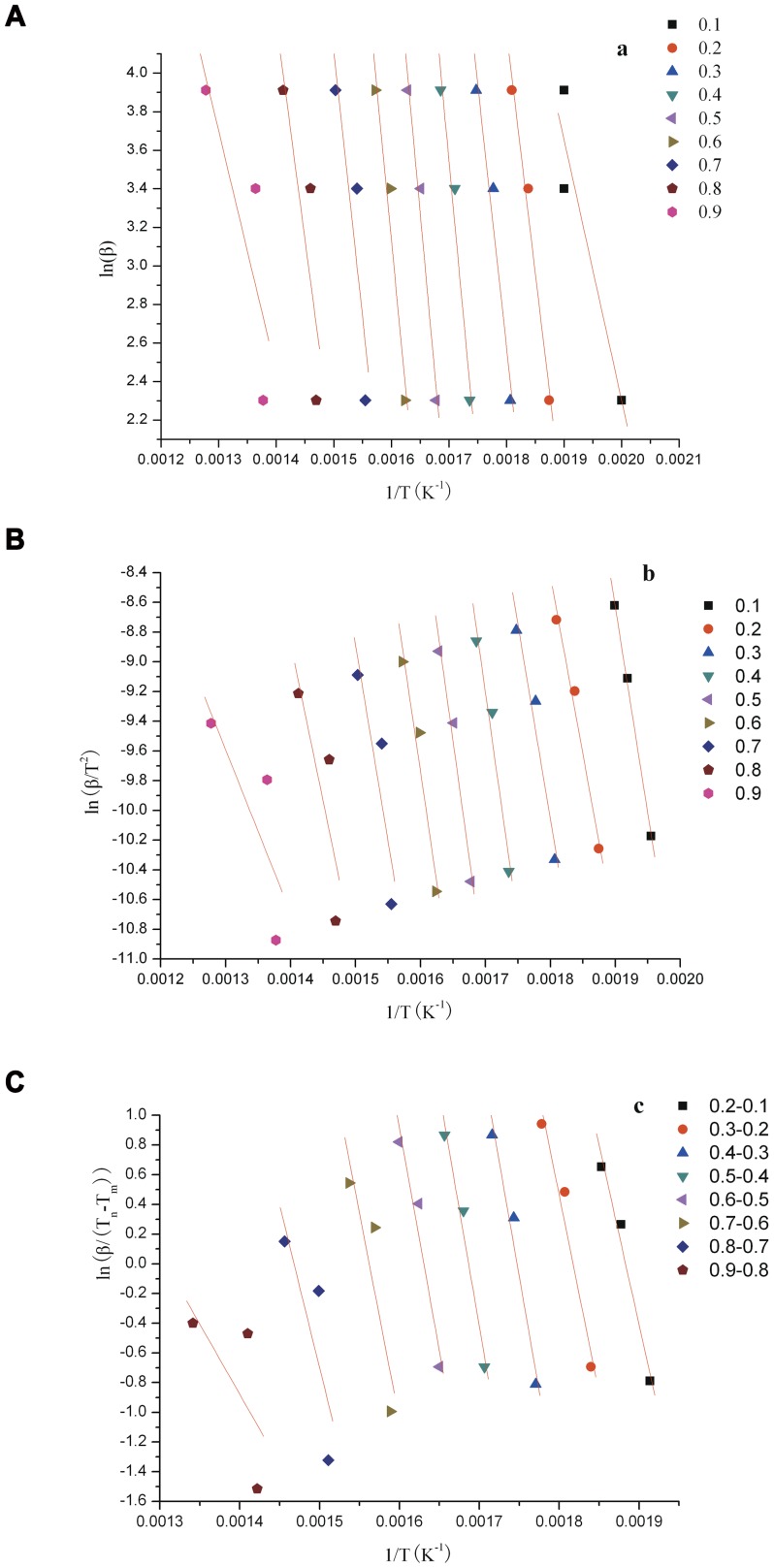
Plots for the activation energies of *Perilla frutescens* polysaccharide in the second stage of pyrolysis.

The activation energy can be determined without confirmation of the reaction mechanism based on the FWO and KAS methods. Temperature integration is not adopted in the Popescu method to avoid the Arrhenius equation and compensation action, among others [27]. Therefore, the mechanism function can be reliably confirmed using the Popescu method. FWO and KAS are adopted to verify the calculation results of the Popescu equation. The linear fitting of the change rates based on the three methods is shown in Figure 3 (a: FWO, b:KAS, c: Popescu), and the kinetic parameters obtained are shown in Table 2. The activation energies calculated from the three methods are similar to the ln*A* value. Therefore, the activation energies calculated by the three methods are generally all effective and similar to one another. However, the activation energy fluctuates under different conversion rates, which can be attributed to the complicated sample composition and complex reaction during pyrolysis. Table 3 compares various kinetic parameters of pyrolysis between different biomass sources [25], [33]–[35]. None of these published results are similar to ours, which suggests that thermal behavior is influenced greatly by the type of feedstock.

**Table 2:** Kinetic parameters obtained by the FWO, KAS, and Popescu techniques at different conversion rates of *Perilla frutescens* polysaccharide.

*A*	FWO			KAS			Popescuc			
	*E* (kJ·mol^−1^)	*r*	ln*A* (min^−1^)	*E* (kJ·mol^−1^)	*r*	ln*A* (min^−1^)	*α*	*E* (kJ·mol^−1^)	*r*	ln*A*(min^−1^)
0.1	228	0.9985	44.8	231	0.9985	45.4	0.2–0.1	201	0.9891	39.4
0.2	198	0.9905	38.5	199	0.9899	38.7	0.3–0.2	221	0.9774	44.1
0.3	216	0.9778	42.8	218	0.9757	43.1	0.4–0.3	257	0.9824	51.0
0.4	254	0.9808	50.5	257	0.9793	51.1	0.5–0.4	261	0.9857	51.2
0.5	258	0.9864	50.7	262	0.9854	51.3	0.6–0.5	250	0.9706	48.2
0.6	246	0.9747	47.7	249	0.9726	48.1	0.7–0.6	231	0.8889	43.8
0.7	219	0.8989	41.6	219	0.8899	41.6	0.8–0.7	182	0.8158	33.7
0.8	179	0.8420	33.2	176	0.8258	32.7	0.9–0.8	79	0.6565	15.4
0.9	99	0.8210	19.2	91	0.7836	17.3				
Average	211			211				210		

**Table 3:** Comparison of various kinetic parameters of pyrolysis for different biomass [25], [33]–[35].

Samples	Cellulose	Hemi-cellulose	Lignin	Chitosan	*Xanthoceras Sorbifolia*polysaccharide
E(kJ·mol^−1^)	200	100	80	95.6–185.7	164

### Determination of the thermal analysis kinetics mechanism

The pyrolysis mechanism is confirmed by the different conversion rates under different heating rates and temperatures. Table 4 shows that the mechanism function is more accurate when the relation coefficient *r* is higher and the standard deviation is smaller [11]. The mechanism function Avrami–Erofeev 20 *g*(*α*) = [−ln(1–*α*)]^4^ is the most suitable for the kinetic mechanism function for the pyrolysis of *P. frutescens* polysaccharide. The core is randomly formed and then predominates in the pyrolysis of *P. frutescens* polysaccharide (*n* = 4). This result can be attributed to the heterogeneous nucleation of the volatile substances caused by the other components within the polysaccharide. This kinetics mechanism is different with that in literature 24. This is probably due to the difference of polysaccharide component and content.

**Table 4:** Linear fitting results of the kinetic mechanism function of *Perilla frutescens* polysaccharide.

Function no.	Temperature (°C)	*r*	SD
20 Avrami-Erofeev Function *n* = 4	220	0.9997	0.0000
	260	0.9995	0.0001
	300	0.9639	0.0105
	340	0.9794	0.1212
	380	0.8751	1.0547

## Conclusions

The pyrolysis of *P. frutescens* polysaccharide can be divided into three stages, namely, the water evaporation of cells, the cracking of the polysaccharide, and the slow decomposition of residues. The heating rate plays a significant role in the pyrolysis of *P. frutescens* polysaccharide. Nitrogen favors the stability and storage of this compound. The Popescu, FWO, and KAS techniques, which yield similar activation energies, are suitable for the determination of the kinetic parameters of *P. frutescens* polysaccharide. The mechanism function Avrami–Erofeev 20 *g*(*α*) = [−ln(1–*α*)]^4^ is the most suitable kinetic mechanism function in the second stage of the pyrolysis of *P. frutescens* polysaccharide.
