# Correction: Pyrolytic and Kinetic Characteristics of the Thermal Decomposition of *Perilla frutescens* Polysaccharide

**DOI:** 10.1371/annotation/f4052f66-23a5-4736-9ad7-b984e6cfb28b

**Published:** 2013-08-08

**Authors:** Quancheng Zhou, Hongmei Zhang, Guihua Sheng

There is an additional author that was not listed on the published article. The following is the correct author and affiliation list:

Quancheng Zhou 1*, Hongmei Zhang 1, Guihua Sheng 2

1. School of Agricultural Engineering and Food Science, Shandong University of Technology, Zibo, China

2. School of Life Science, Shandong University of Technology, Zibo, China

* Corresponding Author e-mail: zhouquancheng@126.com

